# Draft genome sequence of *Petrotoga* sp. DB-2, a thermophilic Fe(III)-reducing bacterium isolated from a petroleum reservoir

**DOI:** 10.1128/mra.01450-25

**Published:** 2026-03-16

**Authors:** Luma Abdulwahed, Anthony C. Greene

**Affiliations:** 1 School of Environment and Science, Nathan Campus, Griffith University, Brisbane, Queensland, Australia; University of Wisconsin-Madison Bacteriology, Madison, Wisconsin, USA

**Keywords:** *Petrotoga*, Fe(III)-reducing bacteria, petroleum reservoir

## Abstract

*Petrotoga* sp. DB-2 is a thermophilic, strictly anaerobic Fe(III)-reducing bacterium isolated from the Fateh oil field, United Arab Emirates. The draft genome is 2,179,419 bp with a GC content of 33.96% and contains 2,141 genes. This genome provides information for future studies of Fe(III) reduction in deep subsurface environments.

## ANNOUNCEMENT

Iron(III)-reducing bacteria are important in anaerobic ecosystems, including petroleum reservoirs, characterized by hydrocarbons, high salinity, and variable temperature and pressure (1, 2). Despite their recognized presence (3), the role of Fe(III) reducers in these environments remains poorly understood compared with other anaerobes like sulfate-reducing bacteria (4). The aim of this work was to understand the molecular mechanisms of Fe(III) reducers and their role in deep subsurface environments.

Strain DB-2 was isolated from a 2-L production water sample collected from the Mishrif reservoir, Fateh oil field (Persian Gulf, UAE; 25°36′47″ N, 54°26′35″ E) in 1996. Enrichment cultures were established by adding 10 mL of water to 90 mL of FRR medium amended with 50 g/L NaCl and incubating anaerobically at 60°C. FRR medium was prepared, as previously described (3). The strain was isolated from a Fe(III)-reducing enrichment by plating onto FRR agar in an anaerobic chamber, as previously described (5). Fe(III) reduction was confirmed using the ferrozine assay (6). Fe(III)-reducing colonies were subcultured and tested for optimal growth temperature and fermentative growth in the absence of external electron acceptors. Cultures were stored at −80°C in 30% glycerol.

For genome sequencing, strain DB-2 was grown from frozen stocks in FRR medium for 72 h at 60°C. Purified DNA was extracted using a Wizard Genomic DNA Purification Kit (A1120; Promega, USA) according to the manufacturer’s instructions. Short read libraries were prepared using the Nextera DNA Flex Library Preparation Kit (20018705; Illumina, USA) and pooled at 2 nM/library. Sequencing was conducted with the NovaSeq 6000 platform using SP Reagent Kit v1.5 (Illumina), and 2 × 150 bp paired-end chemistry. This generated 7,782,290 reads (990 Mbp). Read quality was assessed with FastQC v0.12.0 (7). Default parameters were used for all software unless otherwise specified. Reads were quality trimmed using Trimmomatic v0.36.6 (8) with a 4-bp sliding window and a minimum quality score of Q20, resulting in 5,271,474 high-quality reads. Sequences were assembled with SPAdes v3.15.5 (9). Quast v5.3 (10) and BUSCO v5.8 (11) were used to assess genome assembly quality. Annotation was performed using Prokaryotic Genome Annotation Pipeline (PGAP) v6.10 (12). Coding sequences (CDSs) were predicted by Prodigal 2.6.3 (13). Taxonomic identification was assessed using average nucleotide identity platform Orthologous ANI Tool (OAT) v0.93.1 (14), and digital DNA-DNA hybridization with Type Strain Genome Server (TYGS) (15).

*De novo* assembly of strain DB-2 produced 113 contigs, with a total genome size of 2,179,419 bp. Genomic features are summarized in Table 1. OAT and TYGS suggest that strain DB-2 aligns closest to *Petrotoga mexicana* DSM 14811 (GenBank accession GCA_002895565.1), with an ANI value of 97.7% and a dDDH (d4) of 79.8%, respectively.

**TABLE 1:** Assembly and genomic features of *Petrotoga* sp. DB-2

Feature	Result
Genome size (bp)	2,179,419
Coverage (x)	445
GC content (%)	33.96
Total number of contigs	113
Number of contigs >500 bp	74
Largest contig (bp)	217,676
N50	60,301
L50	11
Total genes	2,141
Coding sequences	2,078
tRNA	48
rRNA	12
Pseudogenes	19
CRISPRs	1

Strain DB-2 grew fermentatively, with optimal growth at 55°C–60°C and reduced Fe(III) in laboratory studies. The genome encodes multiple electron-transfer proteins, including monoheme c-type cytochromes and diverse oxidoreductases that contribute to thermophilic Fe(III) reduction. No orthologs of well-characterized Fe(III) reduction systems found in mesophiles, such as *Shewanella* or *Geobacter*, were detected. Genes associated with fermentative metabolism of acetate, lactate, butyrate, alcohols, and various carbohydrates were identified.

## Data Availability

The genome sequence and annotation data for Petrotoga sp. DB-2 were deposited in DDBJ/ENA/GenBank under BioProject number PRJNA1345258, BioSample number SAMN52666360, and accession number JBSVZD000000000. Raw sequence reads are available in the Sequence Read Archive with accession number SRR36035255.
